# Sanguinarine, Isolated From *Macleaya cordata*, Exhibits Potent Antifungal Efficacy Against *Candida albicans* Through Inhibiting Ergosterol Synthesis

**DOI:** 10.3389/fmicb.2022.908461

**Published:** 2022-06-15

**Authors:** Ziwei Hu, Hao Hu, Zhili Hu, Xiaojun Zhong, Yifu Guan, Yunshi Zhao, Lu Wang, Liang Ye, Liliang Ming, Muhammad Shahid Riaz Rajoka, Zhendan He, Yan Wang, Xun Song

**Affiliations:** ^1^School of Basic Medicine, School of Pharmaceutical Sciences, Respiratory Medicine Department, Shenzhen University General Hospital, Health Science Center, Shenzhen University, Shenzhen, China; ^2^Huazhong University of Science and Technology Union Shenzhen Hospital, Shenzhen, China; ^3^Key Laboratory of Chemistry and Engineering of Forest Products (State Ethnic Affairs Commission), Guangxi Collaborative Innovation Center for Chemistry and Engineering of Forest Products, School of Chemistry and Chemical Engineering, Guangxi Minzu University, Nanning, China; ^4^People's Hospital of Wanning, Wanning, China; ^5^College of Pharmacy, Shenzhen Technology University, Shenzhen, China; ^6^Translational Medicine R&D Center, Shenzhen Institutes of Advanced Technology, Chinese Academy of Sciences, Shenzhen, China

**Keywords:** sanguinarine, antifungal, *C. albicans*, ergosterol, mechanism

## Abstract

In recent decades, infections caused by the opportunistic fungus *Candida albicans* have increased, especially in patients with immunodeficiency. In this study, we investigated the mechanism of action of sanguinarine (SAN) against *C. albicans* both *in vitro* and *in vivo*. SAN exhibited antifungal activity against *C. albicans* clinical isolates, with MICs in the range of 112.8–150.5 μM. Furthermore, scanning electron and transmission electron microscopy showed that SAN induced morphological changes as well as structure disruption in *C. albicans* cells, including masses of cellular debris, ruptured cell walls, and membrane deformation. Flow cytometry revealed that SAN could lead to cell membrane damage, and ergosterol content analysis indicated that SAN could cause ergosterol content reduction exceeding 90%. Further, we validated the efficacy of SAN against candidiasis caused by *C. albicans* in a murine model, and SAN significantly improved survival and reduced weight loss compared to vehicle. The treatment of 1.5 and 2.5 mg/kg/d SAN obviously reduced the fungal burden in the kidney. In addition, histopathological examination indicated that no fungal cells were observed in lung and kidney tissues after SAN treatment. Hence, this study suggests that SAN is a promising plant-derived compound for the development of an effective anticandidal agent.

## Introduction

It has been suggested that antibiotics use, immunosuppressive medication use, implanted medical devices, and immunocompromised medical conditions have all contributed to an upsurge in fungal infections in the last several years (Li et al., 2017). *Candida* species are major human yeast pathogens worldwide, with mortality rates of ~50% (Pfaller and Diekema, 2007; Bassetti et al., 2014). However, toxicity, unwanted side effects, and limited drug absorption have slowed the development of new fungicides (Vandeputte et al., 2012). Throughout human history, medicinal plants have played an essential role in the maintenance of human health. Indeed, medicinal plants have been extensively used as medicines in many Asian countries for more than five thousand years (Yuan et al., 2016), as a source of antibiotics, antineoplastic agents, analgesics, cardioprotective agents, and other biologically active agents (Chen et al., 2015). Recently, it was suggested that ~70–90% of the population of developing countries continue to use traditional medicinal plant medicines to achieve their medical needs (Chen et al., 2015). The most powerful and biologically active agents from plants are their secondary metabolites, which have been extensively explored for the development of novel drugs (Anand et al., 2019). For instance, the discovery of artemisinin, which is derived from *Artemisia annua* L. (sweet wormwood), a shrub used in traditional Chinese medicine, led to Tu You-You receiving the Nobel Prize in Medicine/Physiology in 2015 (Shen et al., 2018). This illustrates the vital role played by plant-derived compounds in pharmacological research for the development of novel, efficient, and safer antifungals.

*Macleaya cordata* is a medicinal plant that belongs to the Papaveraceae family. It is usually found in the southern part of China, where it is used to treat a variety of diseases. *M. cordata* was originally discovered in Bencao Shiyi (where it was used as a supplement to the Materia Medica of the Tang Dynasty); it has multifunctional bioactivities (Zdarilova et al., 2006; Liu et al., 2017). Alkaloids are the main active components in *M. cordata*, which display antimicrobial effects (Zdarilova et al., 2006) against both human and plant fungal pathogens (Li et al., 2017). Therefore, the bioassay-guided isolation of total alkaloids from *M. cordata* was performed, leading to the isolation of the major compound sanguinarine (SAN). SAN is a quaternary benzophenanthridine alkaloid commonly found in plants such as *Sanguinaria canadensis, Poppy fumaria, Chelidonium majus*, and *M. cordata* (Zdarilova et al., 2006). SAN has demonstrated toxicity in rats, but exhibited a low acute oral toxicity (Liu et al., 2020), with an oral LD_50_ in rats of 1,658 mg/kg body weight (b.w.) (Becci et al., 1987). SAN has also demonstrated some cytotoxicity in cancer cells (Och et al., 2019). In this study, no toxicities were found as organic damage when mice were fed 80 mg/kg b.w. of SAN for 10 consecutive days. Given the diverse activities of SAN (Alcantara et al., 2005; Gaziano et al., 2016; Wong-Deyrup et al., 2021), together with the safe daily oral dose of SAN of 5 mg/kg b.w. (Kosina et al., 2004), this compound is still worthy of study.

To the best of our knowledge, very few studies have been conducted investigating the *in vitro* and *in vivo* antifungal mechanisms of action of SAN. Furthermore, no evidence of SAN's antifungal activity against *Candida* has been observed *in vivo*. As a result, we carried out this study in order to investigate the antifungal mechanisms of SAN and subsequently to investigate its therapeutic efficacy against candidiasis.

## Materials and Methods

### Strains, Media, and Chemical Agents

A panel of *C. albicans* strains including nine clinical isolates was collected from Huazhong University of Science and Technology Union Shenzhen Hospital (Shenzhen, China). *C. albicans* (ATCC 10231) was purchased from ATCC to use in this study. A stock solution of all strains was frozen at −80°C and routinely revived on Yeast Malt (YM) agar or YM medium (Sigma, St Louis, MO, USA). Miconazole (MIZ) and fluconazole (FLC) were purchased from Dalian Meilun Biotech Co., Ltd. (Dalian, Liaoning, China). The Annexin V-FITC Apoptosis Detection Kit was purchased from BD Biosciences (San Jose, CA, USA). Drugs and compounds were prepared in DMSO and stored at −20°C.

### Isolation of SAN and Chemical Characterization

*M. cordata* (Willd.) R. Br. was collected from Guizhou province, China. It was authenticated by Mr. Bo Wen from Guizhou Medical University and Professor Yi-Fu Guan from Guangxi Minzu University, China. The voucher samples were deposited in the Engineering Laboratory of Shenzhen Natural Small Molecule Innovative Drugs, Shenzhen University, Shenzhen, China. One kilogram of ground, air-dried, whole plant of *M. cordata* was soaked with 95% ethanol for 2 days (3 × 5 L). The extraction was dried and then resuspended with 5 L of 1% (v/v) sulfuric acid water overnight. The suspension was filtered, and filtrates were alkalized with 10% aqueous NaOH (pH = 10) to precipitate total alkaloids (7 g; 0.7% yield). The crude alkaloids demonstrated 90% inhibition against *C. albicans* at 100 μg/mL. The crude alkaloids (6.5 g) were further subjected to chromatography on silica gel with a mixture of two solvents (petroleum ether/ethyl acetate = 4:1–1:1, v/v) and methanol to afford five fractions for antifungal screening. The active fraction (93% inhibition against *C. albicans* at 100 μg/mL) was further subjected to preparative TLC elution with CHCl_3_/MeOH/acetone (10:0.3:0.3, v/v) to obtain the main compound (red color), which was further purified by Sephadex LH-20, and then to a C-18 reverse-phase column with gradient elution using MeOH/H_2_O to yield compound **1** (680.6 mg). The compound was dissolved in deuterium methanol and transferred into a 5 mm NMR tube. ^1^H-NMR and ^13^C-NMR spectra were acquired on a 400 MHz Bruker Avance III HD spectrometer (Bruker, Germany). Meanwhile, the red powder of compound **1** was dissolved in methanol for subsequent high-resolution mass spectrometry (HRMS, Agilent, USA) detection.

### Microbroth Dilution Assay

The antifungal activity *via* microbroth dilution assay was performed following the previously reported methods by Song et al. (2020) based on CLSI (M38-A) [Clinical and Laboratory Standards Institute (CLSI), 2008]. Briefly, the target compounds were dissolved in DMSO at a stock concentration of 4 mg/mL. MIZ dissolved in DMSO at 1 mg/mL was used as positive control stock solution. Stock solutions of compounds and MIZ were stored at 4°C for further use. Exponentially growing cultures (OD_600_ = 0.03–0.06) of the strains were prepared from overnight cultures. The overnight cultures (OD_600_ = 0.03–0.06) were diluted 1:10 in broth and added to a 96-well plate (195 μL/well). The plates were incubated for 24 h, and then, the inhibition was calculated by subtracting the absorbance of the blank wells, dividing by the average value for the DMSO-only wells, and multiplying by 100. MICs were defined as the SAN concentrations at which 95% of yeasts' growth was inhibited as compared with negative control.

### Filter Disk Assay

For the filter disk assay, *C. albicans* (ATCC 10231) were grown on YM agar plates overnight at 30°C and then transferred to YM broth medium. Then, the cells were collected by centrifugation, washed twice with PBS, and resuspended in PBS at 1 × 10^6^ cells/mL. From the suspended cell solution, 100 μL of each strain was spread onto the surfaces of YM agar plates. After 1 h, 6-mm diameter filter disks containing drugs (20, 40, and 80 μg), or solvent controls, were placed in the desired place of the agar plates. MIZ and DMSO served as positive and negative controls, respectively. The plates were incubated at 30°C, and zones of inhibition were scored after 3 days.

### Scanning Electron Microscopy (SEM)

The samples were taken from the control and SAN treatments (25 and 31.25 μg/mL) for observation of cell morphology under SEM. For that, the cells were collected after 24 h of incubation and then fixed with 2.5% glutaraldehyde for 2–4 h. The samples were further transferred to alcohol with a concentration gradient of 30, 50, 70, and 90% for 15 min, consecutively. The slides were gold-sputtered and visualized in SEM with the magnification of 1000×, 5000×, and 10,000× (Thermo APREO S, USA).

### Transmission Electron Microscopy (TEM)

*C. albicans* cells (1 × 10^5^ cells/mL) were grown with MIZ at 25 μg/mL or SAN at 31.25 μg/mL at 30°C. After 24 h, cells were harvested and washed twice with PBS, fixed overnight in 2.5% glutaraldehyde, and then placed in 1% osmium tetroxide (OsO_4_) in 0.1 M sodium cacodylate buffer (pH 7.4) for 1 h. Samples were then dehydrated in graded acetone and embedded with EPON-812 (Merck, USA). Ultrathin sections were double-stained with 5% uranyl acetate and 5% lead citrate and then examined under a transmission electron microscope (JEOL F200, Tokyo, Japan).

### Flow Cytometry Assay

*C. albicans* cells in stationary phase were refreshed in YM medium and then cultured for 4 h with SAN at 25, 31.25, or 37.5 μg/mL, separately. Then, cells were prepared and analyzed by flow cytometry following previously reported methods (Hao et al., 2013; Jia et al., 2019).

### Quantitation of Ergosterol Content

Exponentially growing *C. albicans* cells were diluted 100-fold with YM medium and exposed to drug treatment. After 48 h of incubation, the cells were harvested and washed with PBS. Cell total sterol was extracted and measured by high-performance liquid chromatography (HPLC) as previously described (Wong-Deyrup et al., 2021).

### Murine Model of *C. albicans* Infection

Animal experiments were approved and performed according to the regulations of the Animal Care and Use Committee of Shenzhen University [SYXK (Yue) 2014-0140]. Six-week-old ICR female mice were immunosuppressed by intraperitoneal (i.p.) injection with cyclophosphamide (CY, 200 mg/kg b.w.; Sigma, USA) in the first 3 days. Mice were then inoculated i.p. with 200 μL of *C. albicans* cells at 1 × 10^8^ cells/mL in normal saline suspension on Day 4 and randomly placed into five groups: model group, a 1.5 mg/kg FLC group, a 0.5 mg/kg SAN group, a 1.5 mg/kg SAN group, and a 2.5 mg/kg SAN group, with 10 mice in each group. Ten uninfected mice were used for the normal control group. At 1 h after infection, each group was administered drug or water *via* oral gavage, and this was repeated for 7 days. The body weight and survival rates of mice were monitored for 10 days.

### Histopathological Assay

On Day 10, mice were killed to collect kidneys and lungs. For the model group, kidney and lung tissues of dead mice were immediately collected for histology. Tissue samples were fixed with fresh 4% (w/v) formaldehyde solution, trimmed, washed, dehydrated, and embedded in paraffin wax, as previously described (Song et al., 2016). The samples were then sectioned and stained with Periodic Schiff-Methenamine Silver (PASM) and hematoxylin–eosin (H&E) for microscopy assays.

### Determination of Fungal Burden

The mice were killed on Day 10, and all organs were aseptically dissected, weighed, homogenized in PBS, serially diluted, and plated on YM agar with streptomycin for counting of colony-forming units (CFUs). The number of colony-forming units could be determined after 48 h growth at 30°C; the results are expressed as CFU/g tissue.

### Statistical Analyses

The data are represented as mean ± SD of three independent experiments. The results were compared using one-way analysis of variance (ANOVA) and Tukey's multiple-comparison post-test. The data analysis was conducted using SPSS statistical software (version 17.0; SPSS Inc., Chicago, Illinois, USA), and the level of statistical significance was set at *p* < 0.05.

## Results

### Isolation of SAN (1)

**Figure 1:**
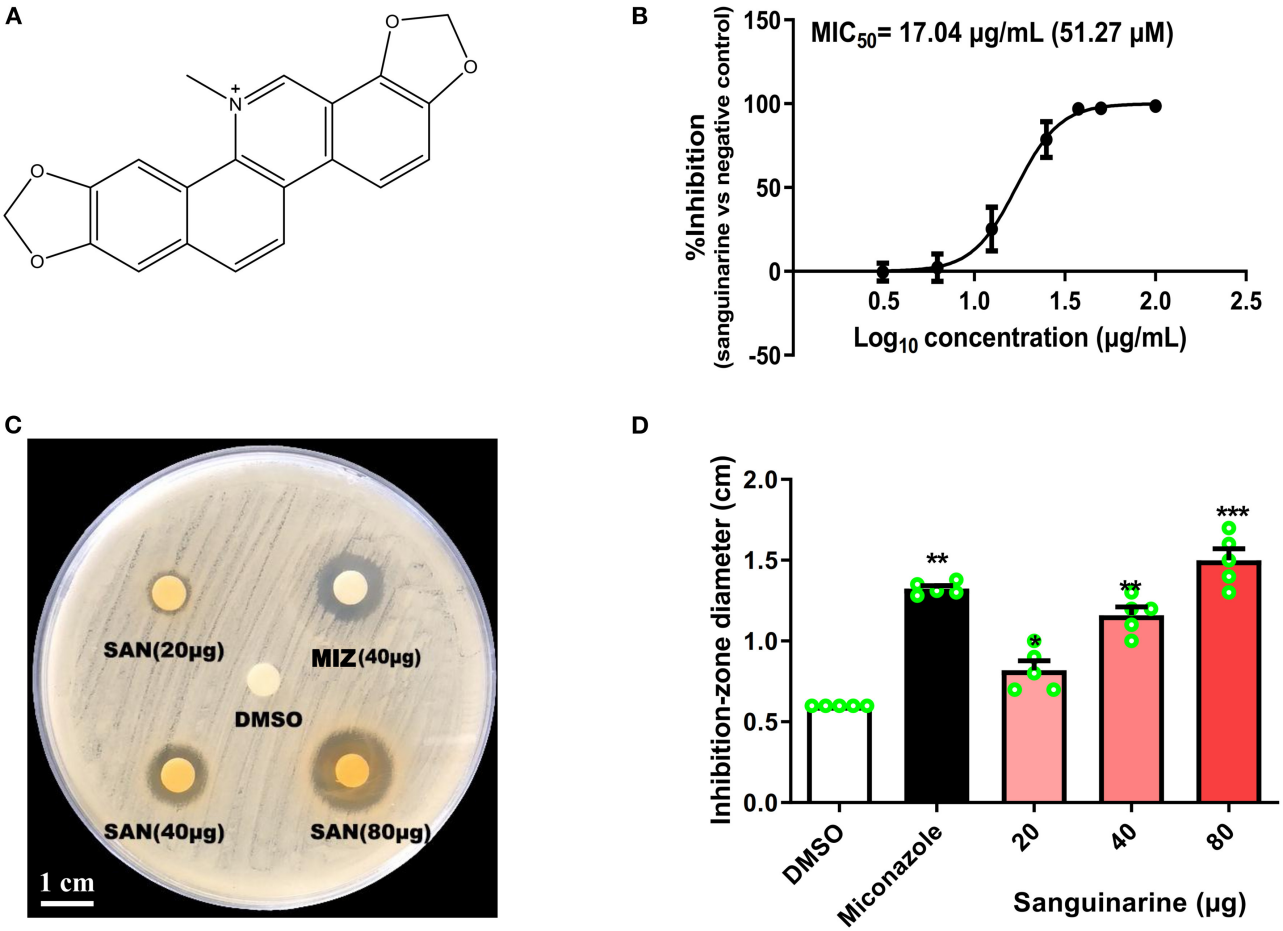
**(A)** Molecular structure of sanguinarine (SAN). **(B)** SAN dose–response curve for inhibitory effects against *C. albicans* (ATCC 10231). A seven-point dose–response relationship was determined for SAN in the antifungal microbroth dilution assay. The data were plotted as percentage of control (100%) at each dose point. The MIC_50_ value was determined at 24 h incubation. **(C)** A representative *C. albicans* petri-dish sample with the zone of inhibition marked. **(D)** Effects of SAN on the inhibition zone diameter produced against *C. albicans*. Miconazole (MIZ, 40 μg) was served as a positive control. Values are expressed as mean ± SD, *n* = 3. *p < 0.05, **p < 0.01 and ***p < 0.001 vs. the DMSO control group.

The total alkaloids from whole plants of *M. cordata* showed antifungal activity against *C. albicans*. Subsequently, 1 kg of dried plant of *M. cordata* was recollected from the same location for bioassay-guided isolation to obtain SAN (**1**) (Figure 1). The molecular formula of **1** was assigned as C_20_H_14_${\text{NO}}_{4}^{+}$ by HRESIMS m/z = 332.0895 (calcd., 332.0917). ^1^H and ^13^C NMR data of **1** clearly showed that it has the basic skeleton of benzophenanthridine alkaloids. The -OCH_2_O- group was positioned between C-7 and C-8 of compound **1**. In addition, **1** showed data similar to those of SAN reported by Marek et al. (1999). Thus, compound **1** was identified as sanguinarine by ^1^H-NMR, ^13^C-NMR, and MS. NMR and MS data are summarized in Supplementary Figures S1–S3 in the Supplementary Materials.

### Antifungal Effects of SAN Against *C. albicans in vitro*

As shown in Figure 1B and Table 1, SAN showed antifungal activity against *C. albicans* and clinical isolates having MICs within the range of 37.5–50 μg/mL (112.8 to 150.5 μM). The comparative efficacy of MIZ was also examined in a disk diffusion assay (Figure 1C). The zone of inhibition of SAN and MIZ against *C. albicans* (mean ± SD) was comparable at 40 μg (Figure 1D).

**Table 1:** Minimum inhibitory concentration (MIC) values (μM) of sanguinarine against *C. albicans* and clinical isolates.

**Strains**	**Source**	**Fluconazole (μM)**	**SAN (μM)**
ATCC 10231	ATCC	≤ 3.3	112.8
CA22	Blood culture	≤ 3.3	112.8
CA24	Blood culture	≤ 3.3	112.8
CA26	Blood culture	≤ 3.3	112.8
CA32	Oral swab	≤ 3.3	150.5
CA34	Oral swab	≤ 3.3	150.5
CA36	Oral swab	≤ 3.3	150.5
CA42	Vaginal swab	≤ 3.3	112.8
CA44	Vaginal swab	≤ 3.3	112.8
CA46	Vaginal swab	≤ 3.3	150.5

### Ultrastructural Analysis Demonstrated the Effective Activity of SAN in *C. albicans*

**Figure 2:**
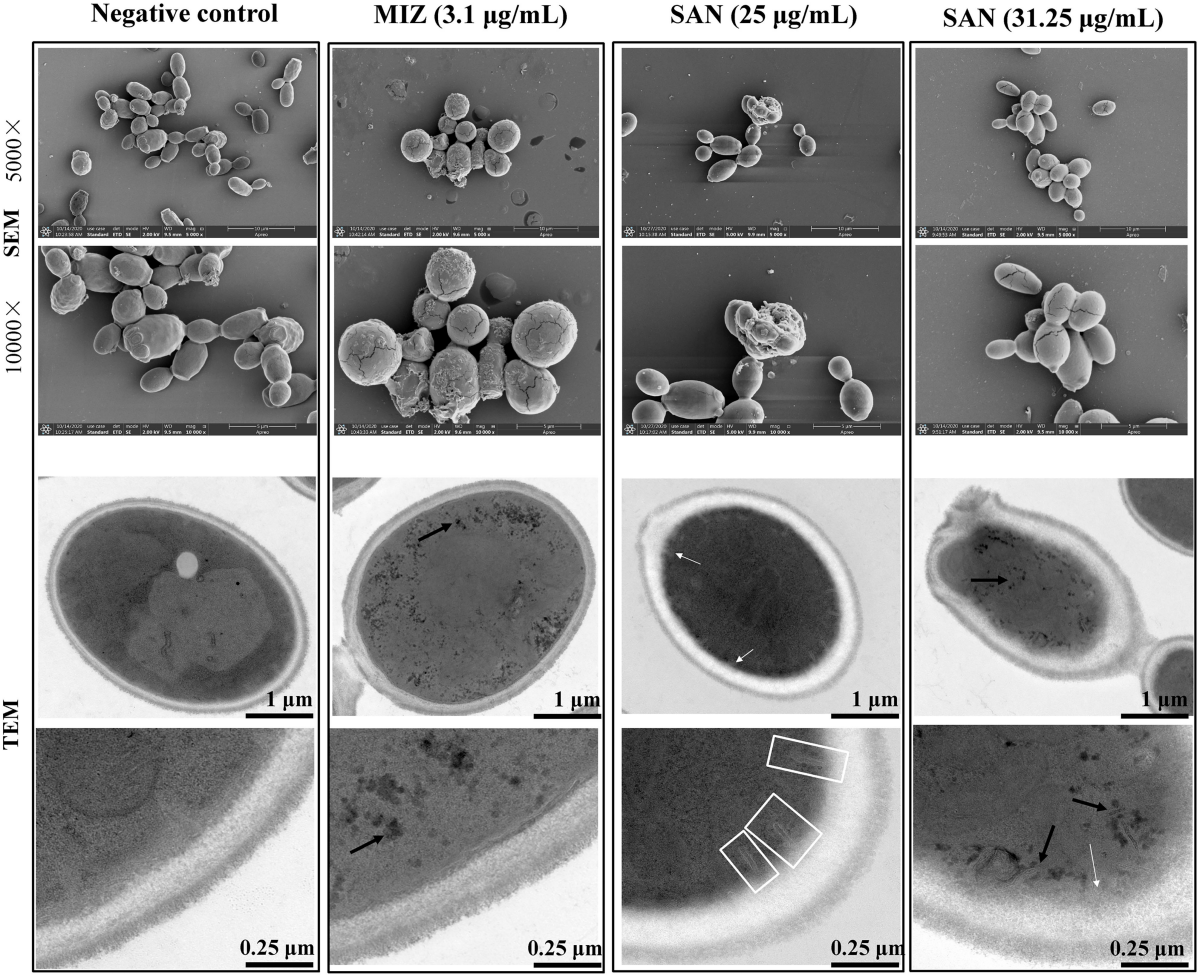
SEM and TEM visualization of the cross sections of the untreated miconazole and SAN-treated *C. albicans* (ATCC 10231) cells after 24 h. Cells were observed under SEM at 5000× and 10000× magnification. TEM showed the changes in cell ultrastructure after incubation with SAN or miconazole including the following: cell showing organelles leakage from vacuole (black arrows), multilayer structures (shown by white arrows) in the plasma membrane, and invaginations of the plasma membrane extending into the cytoplasm (shown by white frames). Three independent experiments, six biological replicates/group, were performed for each drug treatment. Micrographs in this image represent one of six biological replicates analyzed.

Scanning and transmission electron microscopy were used to examine the effect of SAN on the morphology and ultrastructure of *C. albicans*. Our results showed that more than 50% of *C. albicans* cells had noticeable morphological changes after SAN treatment. The untreated *C. albicans* exhibited typical oval morphology, with smooth, homogeneous, and regular cell surfaces (Figure 2). Cells treated with MIZ and SAN had abnormal morphology. Cells treated with SAN [25 μg/mL (75.2 μM) and 31.25 μg/mL (94.0 μM)] were irregular, shrunken, and ruptured in comparison with untreated controls, often with deposits on the cell surface. The cell walls of MIZ- and SAN-treated cells were swollen, and the outer layers were ruptured with a rugged surface or adherent cellular debris. Treated cells had a higher ratio of lysed cells compared to controls, and the number of cells with an irregular surface increased as a function of SAN concentration.

As shown in Figure 2, untreated controls had a well-conserved morphology, with intact cell walls and membranes. A typical *C. albicans* cell wall is composed of an outer electron-dense layer, a low-density intermediate space, and the thin innermost layer of the cell membrane. In normal *Candida* cells, the intra-morphological structure with several organelles is surrounded by the cytoplasm. The plasma membrane and cell wall have a uniform thickness. Exposure of *C. albicans* to MIZ or SAN resulted in significant ultrastructural alterations, such as a shrunken cytoplasmic membrane detached from the cell wall, bulbous structures at the membrane (white arrows), as well as branched and narrow invaginations protruding into the cytoplasm (white frames). Furthermore, mitochondrial structural damage was also observed, especially in intracellular organelles; the integrity of the vacuole was disrupted, as demonstrated by the visible pores and black leakages (black arrows). Some organelles, such as the nucleus and vacuole, disappeared. In addition, impartiality of the cell membrane from the cell wall and pore formation was observed, which resulted in the leakage of cell contents. The leakage of the intracellular contents from the cells was more pronounced, which finally led to cell death. All these abnormal structures reflect damage to the plasma membrane by SAN, suggesting that SAN may penetrate the cell membrane and subsequently interact with cellular organelles.

### Cell Membrane Disruption of *C. albicans* by SAN

**Figure 3:**
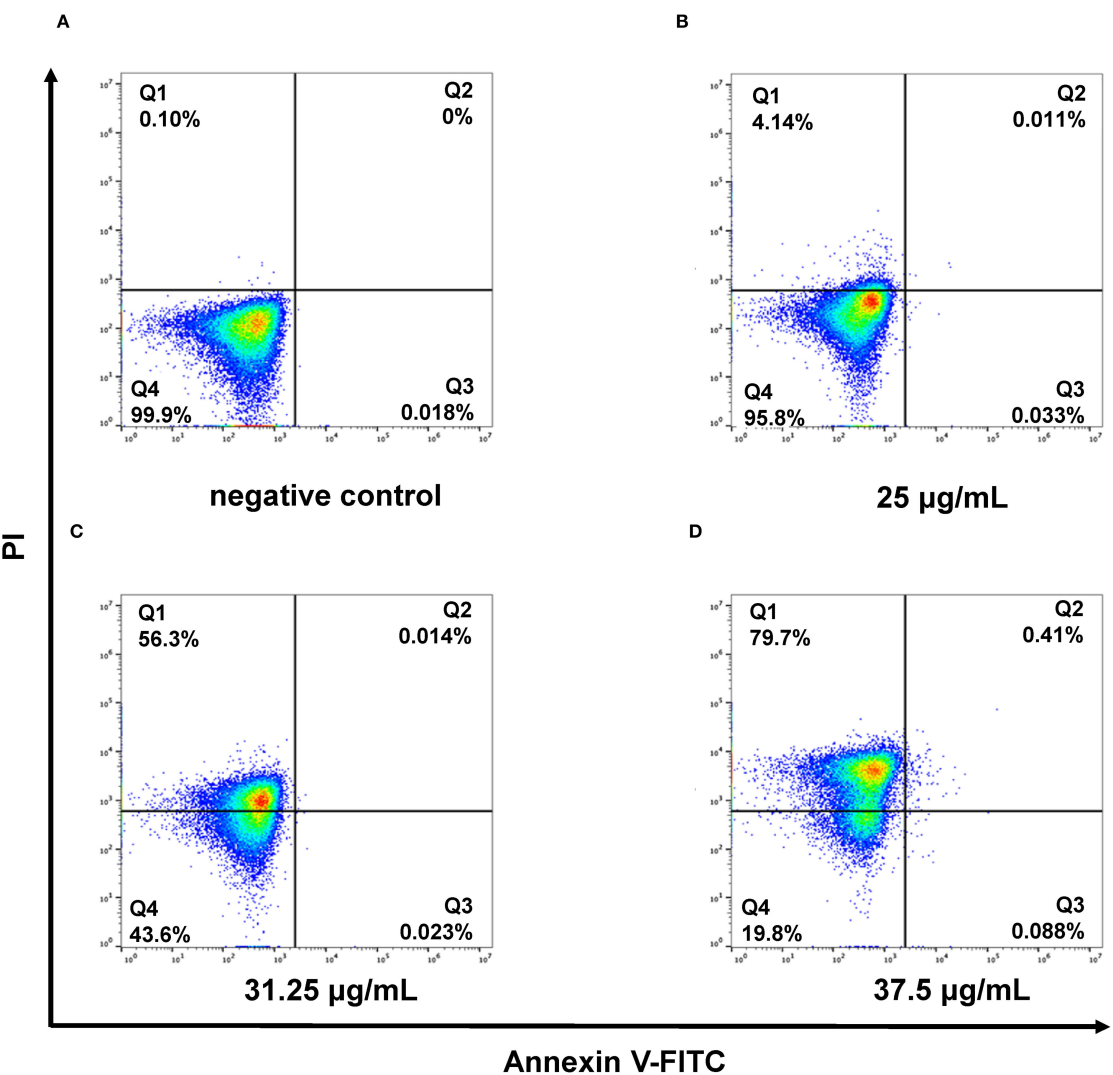
Flow cytometric analysis revealed the necrotic cell death caused by membrane damage in the presence of SAN for 4 h in *C. albicans* (ATCC 10231). Negative control cells without SAN **(A)**, 12.5 μg/mL of SAN-treated cells **(B)**, 31.25 μg/mL of SAN-treated cells **(C)**, and 37.5 μg/mL of SAN-treated cells **(D)**. Q1: necrosis; Q2: late apoptosis; Q3: early apoptosis; and Q4: alive.

The damage of the cell membrane due to SAN treatment was further confirmed by the analysis of phosphatidylserine externalization in *C. albicans* using flow cytometry. The interaction of SAN with *C. albicans* was observed to cause more noticeable cell membrane damage in the yeast, as an increase in PI fluorescent signal was observed (shift toward the upper-left quadrant, the FITC-/PI+). In Figure 3, a significant, dose-dependent increase in *C. albicans* cells stained by PI can be seen after 4 h of SAN treatment (4.14, 56.3, and 79.7%) compared with the negative control (0.1%), suggesting that SAN affected cell membrane integrity in *C. albicans* cells.

### Assessment of Ergosterol Content

**Figure 4:**
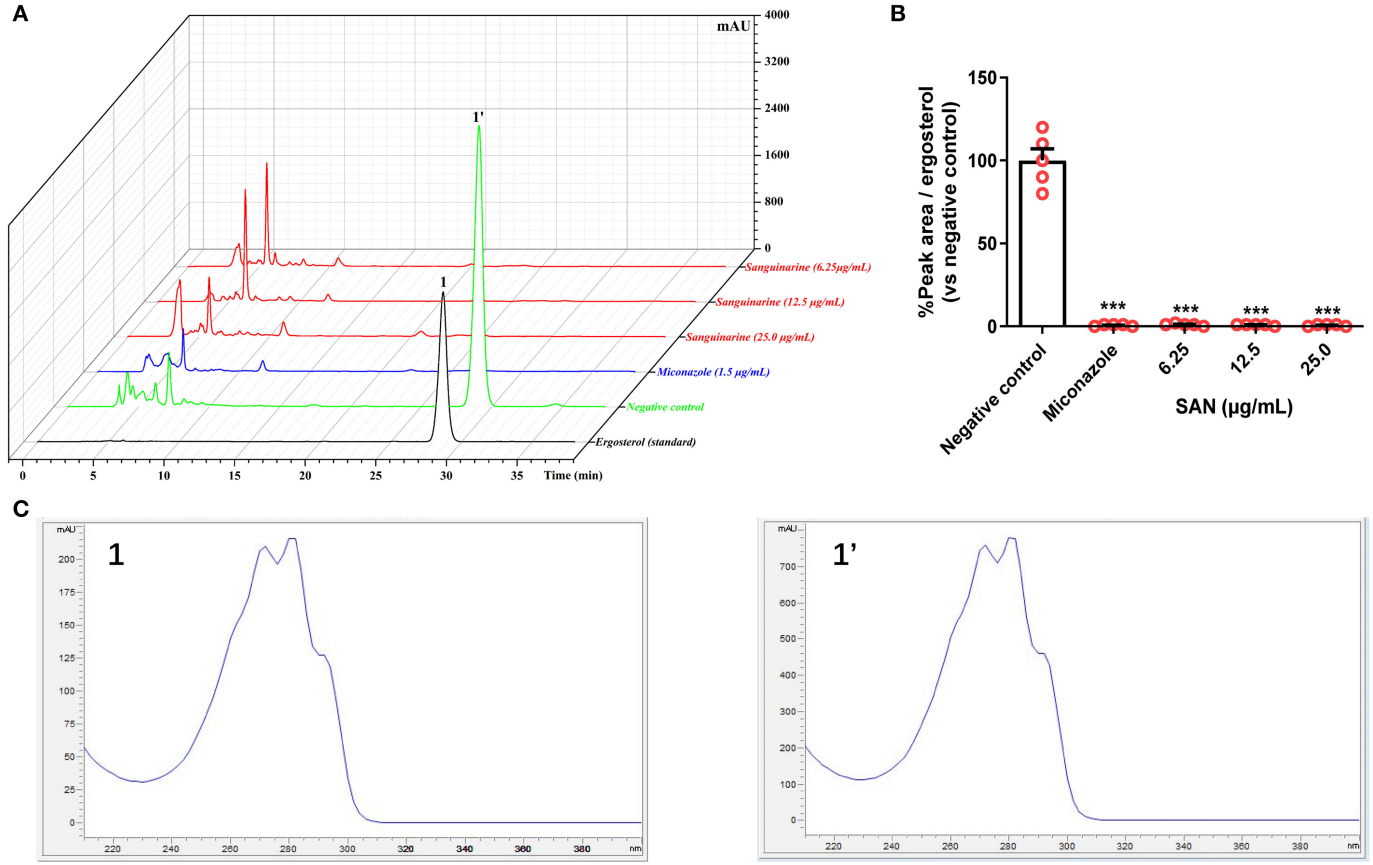
**(A)** Superimposed HPLC-DAD chromatograms at 282 nm of ergosterol extracted from *C. albicans* treated with miconazole (1.5 μg/mL) and SAN (6.25, 12.5, and 25.0 μg/mL). **(B)** Relative HPLC peak areas of ergosterol [Retention time (RT) = 28.7 min]. **(C)** The comparison of UV Spectra data between the peak 1 (standard ergosterol) and 1' at RT = 28.7 min. The data are presented as the mean ± SEM (*n* = 4, ***p < 0.001 compared to negative control, ANOVA).

Previous docking simulations evidenced a high affinity and tight binding capacity of SAN toward the active site of CaCYP51, with the binding affinity of −11.6 kcal/mol (Wong-Deyrup et al., 2021). The residues around SAN are hydrophobic environments of Phe126 and Phe228. The five-membered heterocyclic rings with two oxygens in SAN mediated hydrogen bonding with the hydroxyls of Tyr132, Phe233, and Gly307 (Wong-Deyrup et al., 2021). Those findings indicated that SAN may be an inhibitor of CaCYP51, which could result in a decrease in ergosterol expression. Thus, ergosterol contents were extracted from the cells in control, MIZ, and SAN after 48 h treatment. The ergosterol retention time was 28.7 min in HPLC (Figure 4A), which was identified by ergosterol standard and UV spectrum (Figure 4C). SAN and MIZ treatment of *C. albicans* resulted in an ergosterol content reduction above 95% (*p* < 0.05, Figure 4B), in comparison with the negative control. These results indicate that SAN caused aberrations in sterol biosynthesis, leading to the lack of ergosterol in the membrane of *C. albicans*.

### SAN Administration Increased Survival Time and Body Weight After *C. albicans* Infection

**Figure 5:**
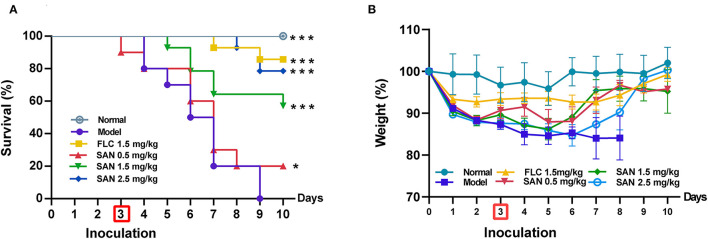
Survival **(A)** and body weight change **(B)** of immunocompromised mice infected with *C. albicans* at 1× 10^8^ CFU/mL. Animals were treated for 10 days with water (normal group without infection; infection model group), fluconazole (FLC, 1.5 mg/kg/d), and SAN (0.5, 1.5, and 2.5 mg/kg/d, respectively). The results are displayed as means ± SEM from three independent experiments. *p < 0.05/***p < 0.001 vs. model.

The *in vivo* therapeutic efficacy of SAN was evaluated by a standard CY-induced immunosuppression murine model of disseminated candidiasis (Andes, 2005). In Figure 5A, sham-treated mice that received sterile water all died 6 days after infection, while the group administrated 1.5 mg/kg FLC showed 80% survival within 10 days. SAN therapy at 1.5 mg/kg resulted in an improvement in survival of 70%. The survival of the group treated with 0.5 mg/kg SAN was 20% on Day 10. In addition, *C. albicans* infection led to a 15% loss of body weight in mice after 3 days of infection. In both groups of mice, SAN-administrated mice experienced a rapid increase in weight on Day 5 and had less body weight loss at the 10-day mark in comparison with controls (<10%, Figure 5B).

### Reduction in Kidney Fungal Burden on *in vivo* Treatment With SAN

**Figure 6:**
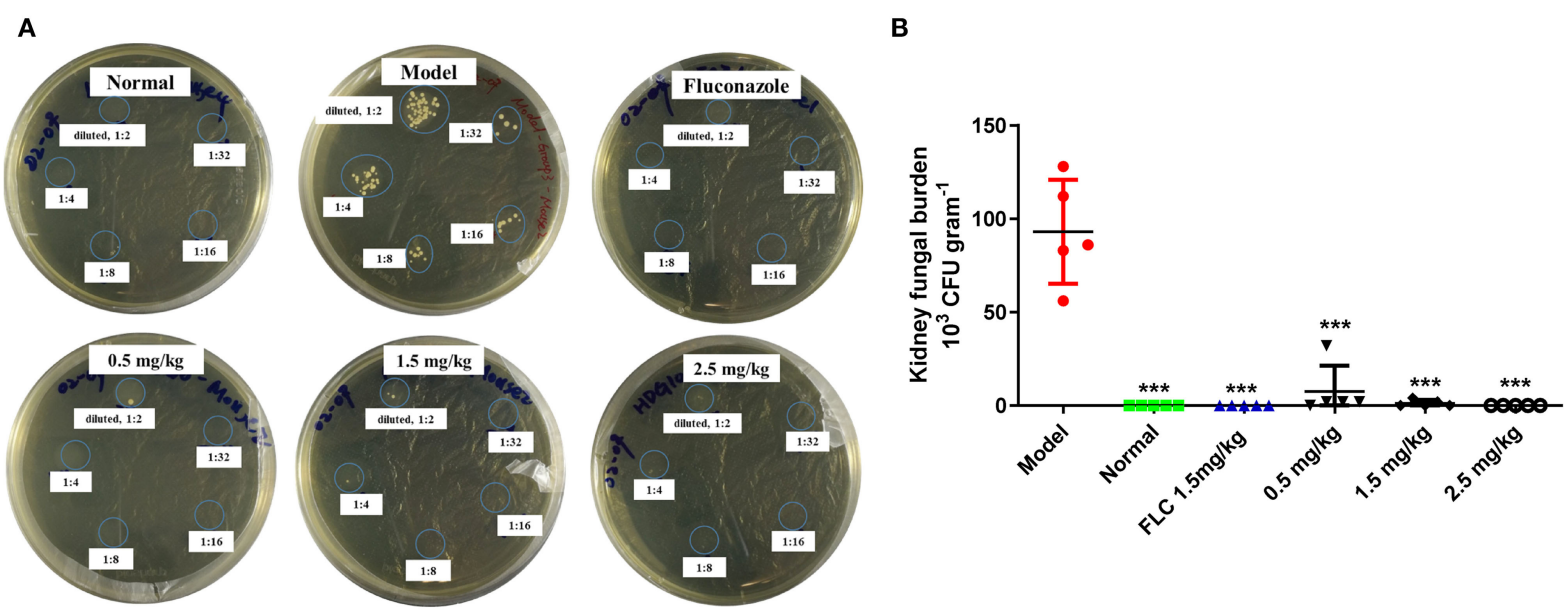
Fungal burden in the kidneys. **(A)** *C. albicans* CFUs in the kidney tissue at killing are shown in panels for the representative mice in each group. **(B)** Fungal burdens per gram of the kidney (*n* = 5 mice). Significance is represented with asterisks (***p < 0.001). Normal: no inoculation of *C. albicans*, water-shamed; Model: infected with *C. albicans*, water-shamed; Fluconazole (1.5 mg/kg/d) and SAN (0.5, 1.5, and 2.5 mg/kg/d, respectively).

Water-shammed mice exhibited 9.30 × 10^4^ CFU g^−1^ kidney on average, while no colonies were observed in the groups treated with FLC and 2.5 mg/kg of SAN (Figure 6A). Figure 6B shows that even 0.5 mg/kg of SAN was sufficient to reduce viable *Candida* to 7.60 × 10^3^ CFU g^−1^; 2.5 mg/kg of SAN led to a dramatic reduction in fungal burden in the kidney, indicating its antifungal activity *in vivo*.

### Histological Analysis

**Figure 7:**
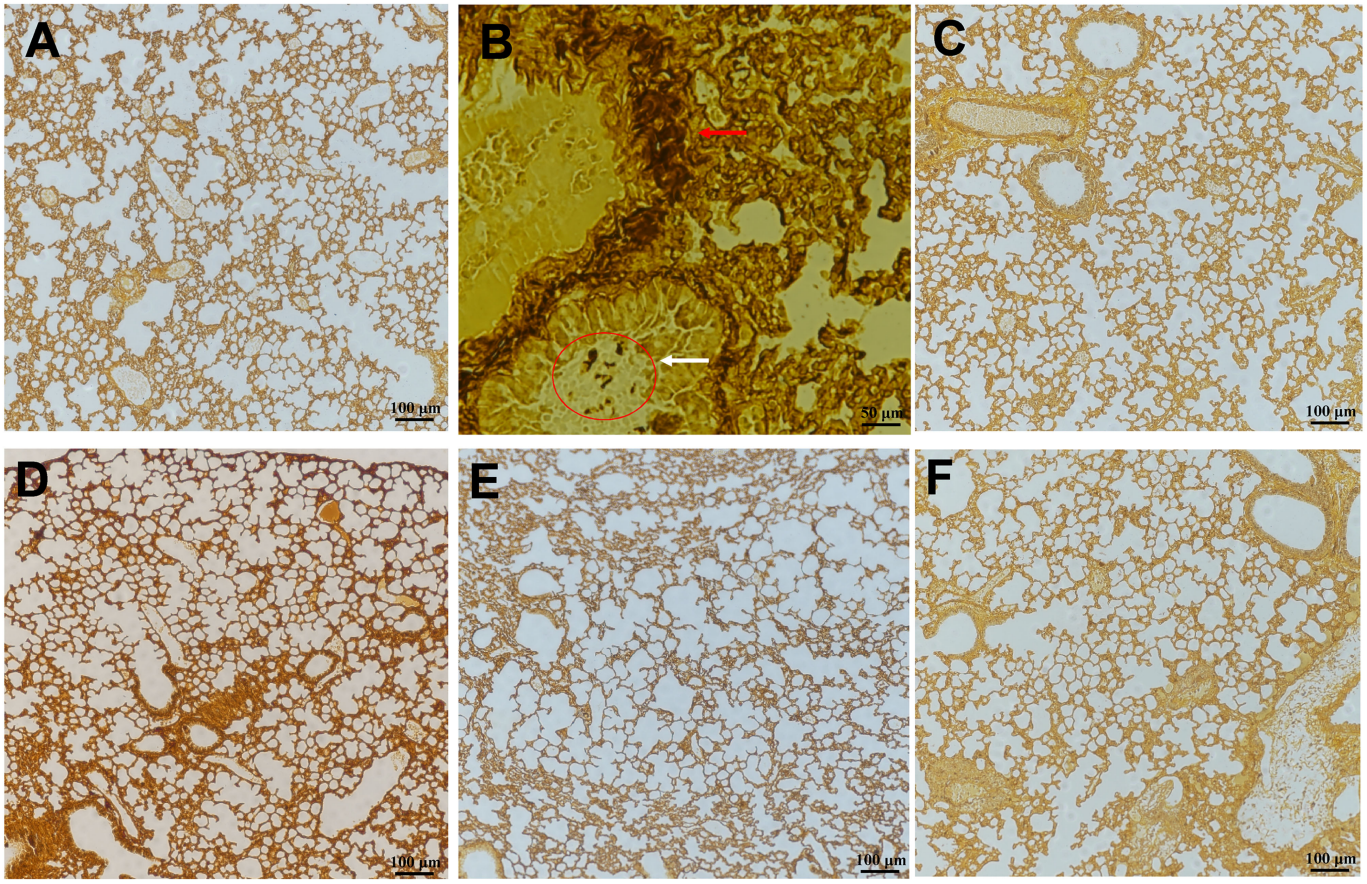
Representative PASM-stained sections of the lungs of mice in various groups after treatment. **(A)** Normal lung section (×200, mice without infection). **(B)** Lung section of *C. albicans*-infected mice treated with water showed numerous intracellular and extracellular, round, oval or elongate, elongated, and moniliform hyphal *C. albicans* (red arrows, ×400). **(C)** Lung section of mice treated with FLC (1.5 mg/kg/d) showed similar findings to uninfected mice (×200). **(D)** Lung section of mice treated with low dosage of SAN (0.5 mg/kg/d), middle dosage of SAN [**(E)**, 1.5 mg/kg/d], and high dosage of SAN [**(F)**, 2.5 mg/kg/d] after PASM staining showed significantly reduced the amount of fungi (×200).

The representative photographs of lungs by PASM staining demonstrate that the uninfected mice had a well-defined and normal lung tissue structure, whereas the water-shammed mice showed an obviously widened alveolar interval, collapsed pulmonary alveolus fusion, and disorganized structure, associated with numerous intracellular and extracellular, oval-to-elongated, thin-walled, yeast-like fungi (Figure 7). Interestingly, PASM analysis indicated a minor histological change in the lung tissues of mice treated with 0.5 mg/kg of SAN, and no fungi were observed in the FLC, 1.5 mg/kg SAN, or 2.5 mg/kg SAN groups, indicating that fungal invasions were dramatically improved.

**Figure 8:**
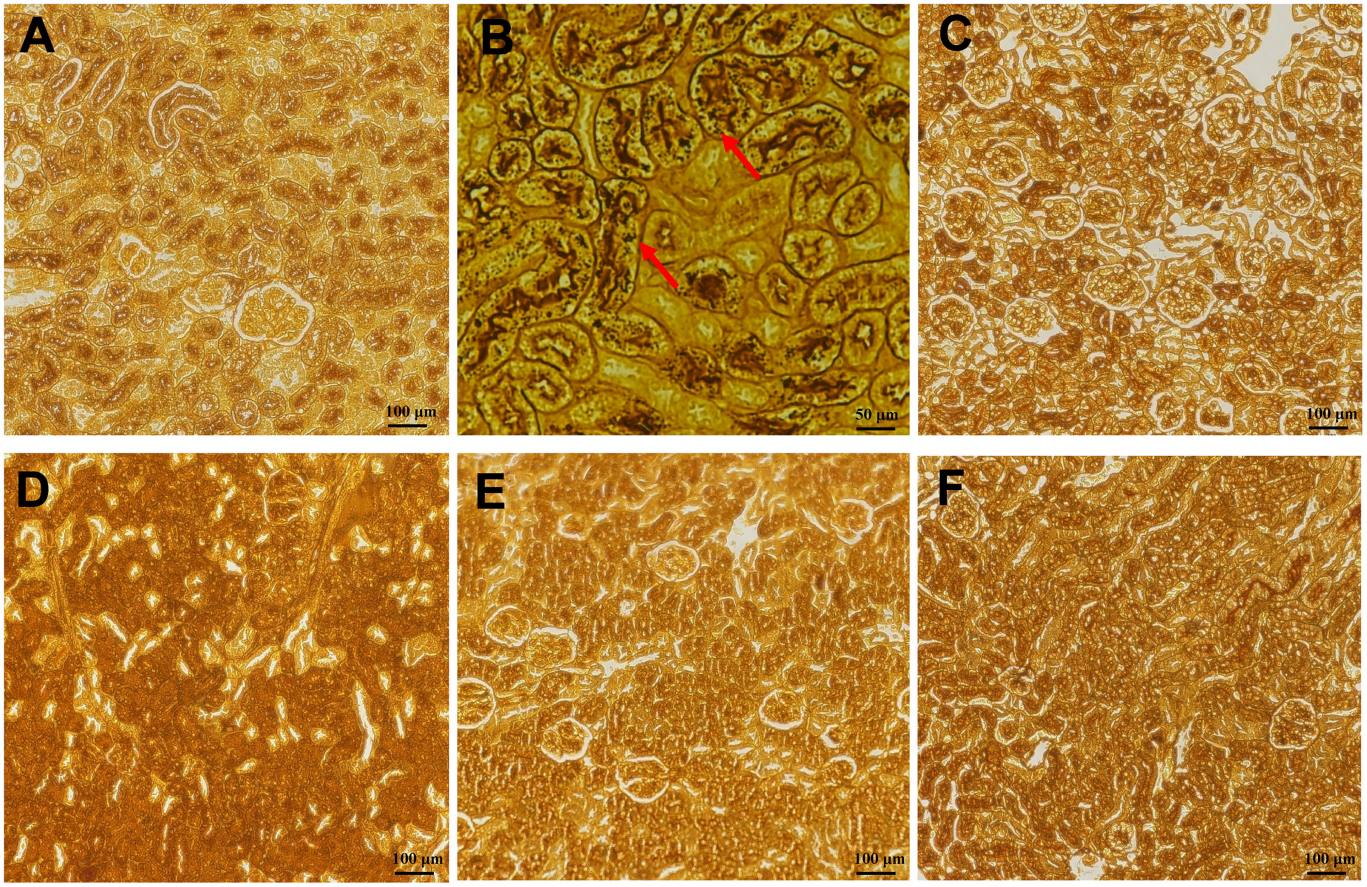
Histopathology of the kidneys. **(A)** Represented the kidneys of normal mice without infection. PASM-stained images are shownfor the infected mice with water **(B)**, or 1.5 mg/kg/d of FLC **(C)**, and the different dosages of SAN [**(D)**, 0.5 mg/kg/d; **(E)**, 1.5 mg/kg/d; **(F)**, 2.5 mg/kg/d], magnifications, ×200. Black staining (red arrows) reflects areas of numerous intracellular and extracellular *C. albicans* cells [**(B)**, ×400].

Compared with normal mice without infection (Figure 8A), kidney sections in water-shammed mice displayed noticeable tissue damage and intense yeasts and hyphae in the proximal and distal convoluted tubule (Figure 8B), which were not observed from kidney sections of infected mice treated with FLC, 1.5 mg/kg, or 2.5 mg/kg of SAN (Figures 8C–F). Meanwhile, in the groups treated with 0.5 mg/kg SAN, *C. albicans* cells were significantly reduced and barely visible by PASM staining, which was consistent with the above-described significant reduction in fungal burden.

**Figure 9:**
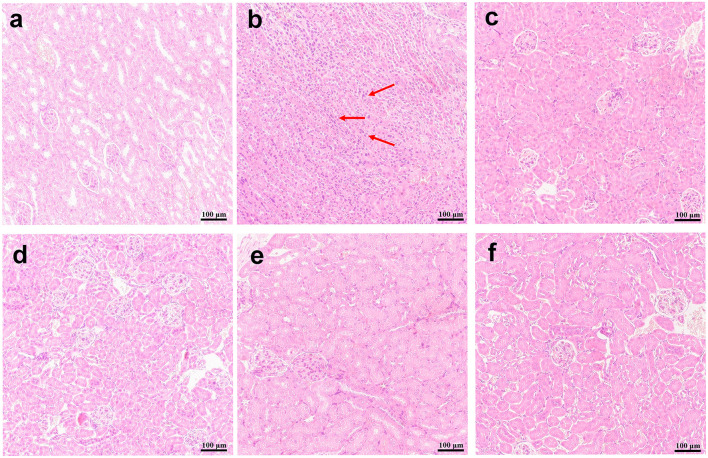
Kidney histopathology. H&E stain representations are provided for **(A)** the kidney of normal mice; **(B)** infections with *C. albicans* alone, or **(C)** treatments with 1.5 mg/kg/d of FLC, **(D)** 0.5 mg/kg/d of SAN, **(E)**1.5 mg/kg/d of SAN, and **(F)** 2.5 mg/kg/d of SAN. Dark blue staining reflects areas of acute inflammation. Magnifications, ×200.

In addition, HE staining of the kidneys from the mice in the infection model group demonstrated gross pathological changes, including the destruction of tissue organization and the massive infiltration of inflammatory cells, while such gross abnormalities were absent in mice in the FLC and SAN treatment groups (Figure 9). Together, our data demonstrated for the first time that SAN appears capable of reducing the fungal invasion of organs in the body.

## Discussion

Invasive candidiasis (IC) is a fungal infection caused by different types of *Candida* yeast, and it is associated with considerable morbidity and mortality globally (Pappas et al., 2018). Among *Candida* species, *C. albicans* is the most frequently isolated fungal species in China (Pfaller and Diekema, 2007). Although azoles have been used in the treatment of IC, their wide use is limited by severe side effects such as nephrotoxicity, hepatotoxicity, and neurotoxicity (Kontoyiannis and Lewis, 2015). Moreover, the increasing multidrug-resistant fungal strains pose a well-recognized global health threat that demands novel alternative fungicidal drugs that are safe and more effective (Srinivasan et al., 2014; Nett and Andes, 2016).

Plant-derived bioactive secondary metabolites provide considerable antifungal drug candidates (Cragg and Newman, 2013). The use of ethnobotanical medicines is accelerating and improving the channel of drug development. Previous research reported that *M. cordata* alkaloids were considered promising sources of antifungal agents against *T. rubrum* and phytopathogenic fungi (Yang et al., 2012; Wong-Deyrup et al., 2021). We confirmed that SAN was one of the active constituents through the bioassay-guided isolation of total alkaloids. SAN exhibited toxicity to mammalian cells, but it is relatively safe to mammalian cells (Zhong et al., 2017). We previously reported that SAN exerted exciting antifungal activities against *T. rubrum via* the inhibition of ergosterol synthesis (Wong-Deyrup et al., 2021). Zhong and Qian et al. found that SAN had inhibitory effects on the formation of *C. albicans* biofilms (Zhong et al., 2017; Qian et al., 2020). The possible mechanism is that SAN inhibits adhesion as well as hyphal and biofilm formation *via* cAMP signaling suppression (Qian et al., 2020). However, the modes of SAN's antifungal action against *C. albicans* are seldom reported.

In this study, we found that SAN was effective in inhibiting the growth of *C. albicans* both *in vitro* and *in vivo*. Our results are consistent with previous research about the antibiofilm effects of SAN against *C. albicans* (Zhong et al., 2017; Qian et al., 2020). Unlike those studies, we confirmed a potential antifungal activity of SAN against *C. albicans* clinical isolates. In line with the previously observed antibiofilm effects of SAN (Zhong et al., 2017; Qian et al., 2021), SAN caused obvious morphological changes in planktonic cells, including cell disruption and changes to the alignment of plasma membranes (Figure 2), demonstrating for the first time that SAN may lead to cell membrane damage. Our results demonstrated that the treatment of *C. albicans* cells with SAN resulted in significant modifications in the cell wall and membrane, as examined by SEM and TEM. Consistent with these observations, SAN may trigger major alterations in *C. albicans* cells, such as a rough wrinkled outer cell wall, ruptured cell membrane, and dense cytoplasm without distinguished features. Therefore, the mechanism of SAN's action on *C. albicans* may be attributed to the disruption of the cell wall and cell membrane, as our results displayed severe modifications to the cell wall, causing alterations on the surface and cell collapse.

In order to confirm that the cell membrane damage was caused by SAN, the fluorescent probe PI was utilized because of its capability to enter into the cytoplasm and bind to DNA (Yang et al., 2019). Flow cytometry analysis showed that SAN caused an apparent increase in PI signals in *C. albicans* cells (Figure 3), indicating that SAN induced cell membrane damage, which is consistent with the observation of disrupted cells treated by SAN under TEM.

Ergosterol is an essential structural component fungal cell membranes, and it plays an important role in maintaining cell integrity (Jordá and Puig; Rodrigues, 2018). Thus, the investigation of vulnerable components in the ergosterol biosynthesis pathway contributed to the development of antifungal agents such as azoles and polyene antifungal agents (Ghannoum and Rice, 1999). Sterol 14α-demethylase (CYP51) is one of the most important targets for developing antifungal drugs to inhibit ergosterol biosynthesis (Becher and Wirsel, 2012; Rosam et al., 2021). In our previous study, the active sites of CaCYP51 were occupied by SAN based on docking (Wong-Deyrup et al., 2021), suggesting that SAN may inhibit the expression of ergosterol catalyzed by CYP51. The ergosterol contents of *C. albicans* at sub-MICs of SAN showed a dose-dependent reduction of above 98% versus the negative control group, as expected. SEM and TEM results demonstrated that SAN can cause damage to the membrane of *C. albicans*, resulting in organelle disorganization in the cytoplasm. These results suggested that SAN probably interferes with the expression of ergosterol-biosynthesis-related CYP51 activity, decreasing ergosterol content, and destroying *C. albicans* cells.

The potent *in vitro* antifungal activity of SAN, its water solubility, and its acceptable oral toxicity (Lu et al., 2012) make it worthwhile to investigate the therapeutic implications of SAN *in vivo*. The intravenous inoculation of *C. albicans* resulted in acute systemic dissemination affecting the deep organs (Conti et al., 2014), and the main target organs were the kidney and lung (Leavy, 2014). Thus, the chemotherapeutic efficacy of SAN was assessed by histopathology of the kidney and lung. We found that SAN treatment at concentrations of 1.5 and 2.5 mg/kg was as effective against disseminated fungal infection as FLC, without severe side effects. Histological staining of lungs showed that SAN did not alleviate pulmonary interstitial edema or inflammatory cell infiltration, nor did it reduce the amount of fungi in the kidney.

Consistent with the histopathological examination, *Candida* colonies were detectable in the tissue cultures from the model and low-dosage groups (Figure 6). Compared with the model group, the 1.5 and 2.5 mg/kg SAN treatments obviously reduced the fungal burden in the kidney and significantly improved the survival rates and body weights. All results indicate that SAN is able to reduce pathogenic *C. albicans* cells in the deep organs of infected mice. Thus, the *in vivo* findings support the antifungal efficacy of SAN observed *in vitro*.

In summary, the available evidence conclusively demonstrates the potential of SAN as a fungicide against *C. albicans*. However, additional detailed investigation on target confirmation is encouraged to establish the use of SAN for candidiasis in future.

## Data Availability Statement

The original contributions presented in the study are included in the article/Supplementary Materials, further inquiries can be directed to the corresponding author/s.

## Ethics Statement

The animal study was reviewed and approved by the Animal Care and Use Committee of Shenzhen University.

## Author Contributions

All authors listed have made a substantial, direct, and intellectual contribution to the work and approved it for publication.

## Funding

This work was supported by Guangdong Basic and Applied Basic Research Foundation (2020A1515111169, 2019B1515120029, 2020A1515110410, 2020A1515011342, and 2021A1515010917), the University Stability Support Program of Shenzhen (20200813201847001 and 20200803131335002), the Shenzhen Nanshan District Scientific Research Program (NS2021105), the Natural Science Foundation of the Education Department of Guangdong Province (2020KZDZX1172), Shenzhen Peacock Plan Project (RC00325, 827/000569, and 827/000655), SZU Top Ranking Project (860000002110131 and 86000000210), the National Natural Science Foundation of China (NSFC, 32170937, 31670360, 81903875, 82174033, and U1702286), and the Science and Technology Program of Shenzhen (JCYJ20190808122213241 and JCYJ20190807160601672).

## Conflict of Interest

The authors declare that the research was conducted in the absence of any commercial or financial relationships that could be construed as a potential conflict of interest.

## Publisher's Note

All claims expressed in this article are solely those of the authors and do not necessarily represent those of their affiliated organizations, or those of the publisher, the editors and the reviewers. Any product that may be evaluated in this article, or claim that may be made by its manufacturer, is not guaranteed or endorsed by the publisher.
