# Supplementary material for: Sanguinarine, Isolated From Macleaya cordata, Exhibits Potent Antifungal Efficacy Against Candida albicans Through Inhibiting Ergosterol Synthesis
Source: Front Microbiol. 2022 Jun 15;13:908461. doi: 10.3389/fmicb.2022.908461 (PMC9240711; doi:10.3389/fmicb.2022.908461)
Supplement: Supplementary file 1 [file Data_Sheet_1.PDF]

Supplementary data

Sanguinarine (**1**): orange-red crystal; ^{1}H-NMR (400MHz, MeOD) δ: 9.95 (1H, s, H-6), 8.64 (1H, d, J =8.9 Hz, H-11), 8.55 (1H, d, J= 8.7 Hz, H-10), 8.23 (1H, d, J = 8.9 Hz, H-12), 8.17 (1H, s, H-4), 7.97 (1H, d, J =8.8 Hz, H-9), 7.58 (1H, s, H-1), 6.55 (2H, s, 1,2-OCH_2O), 6.29 (2H, s, 7,8-OCH_2O) and 4.97 (3H, s, NCH_3); ^{13}C-NMR (100 MHz, MeOD) δ: 105.6 (C-1), 149.4 (C-2), 149.3 (C-3), 103.6 (C-4), 119.9 (C-4a), 131.5 (C-4b), 149.5 (C-6), 109.9 (C-6a), 146.8 (C-7), 148.1 (C-8), 120.6 (C-9), 116.9 (C-10), 127.7 (C-10a), 126.2 (C-10b), 118.2 (C-11), 131.5 (C-12), 132.8 (C-12a), 102.9 (2,3-OCH_2O), 105.1 (7,8-OCH_2O) and 51.4 (N–CH_3). ESI–MS (+) m/z 332.0895 $[M]^+$. NMR and MS data of **1** are displayed in Figure S1, S2 and S3.

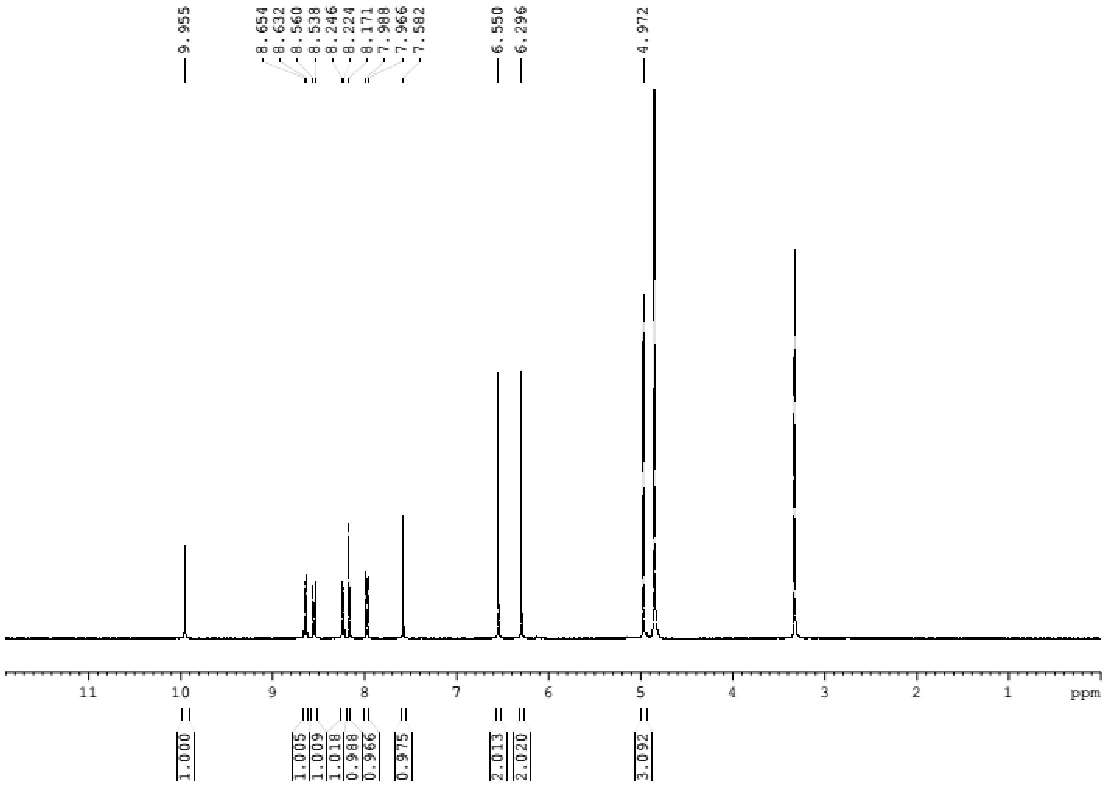

Figure S1 ^{1}H NMR (400 MHz, MeOD) spectrum of compound **1**

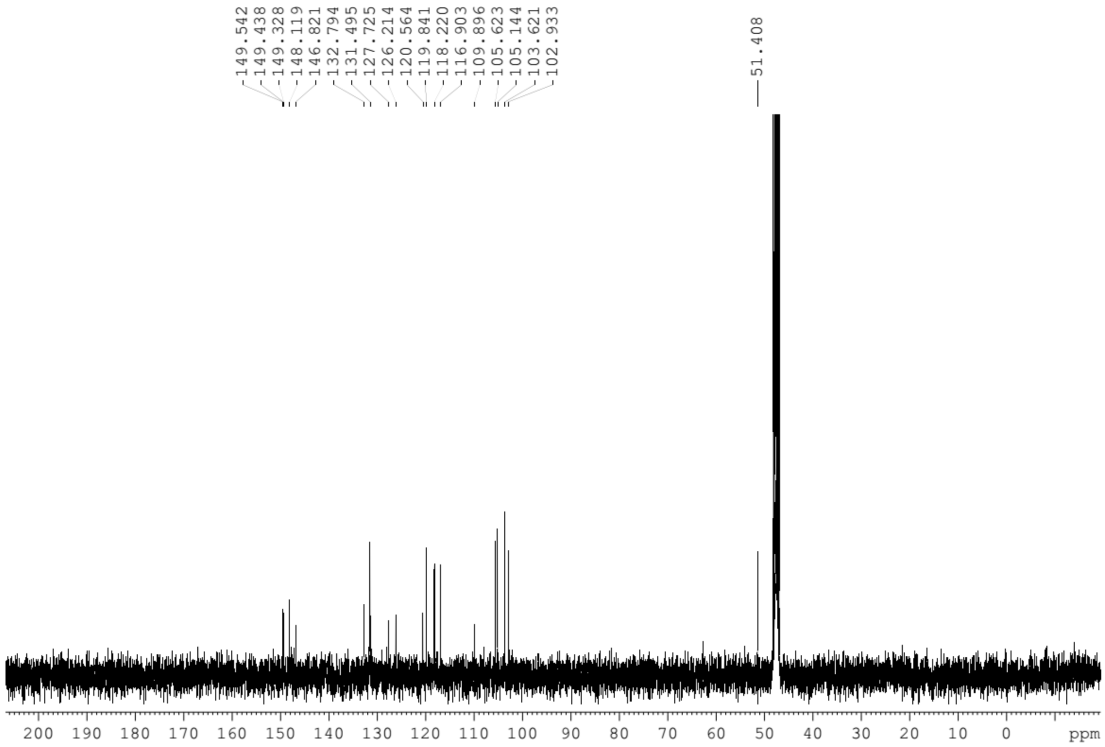

Figure S2 ^{13}C NMR (100 MHz, MeOD) spectrum of compound **1**

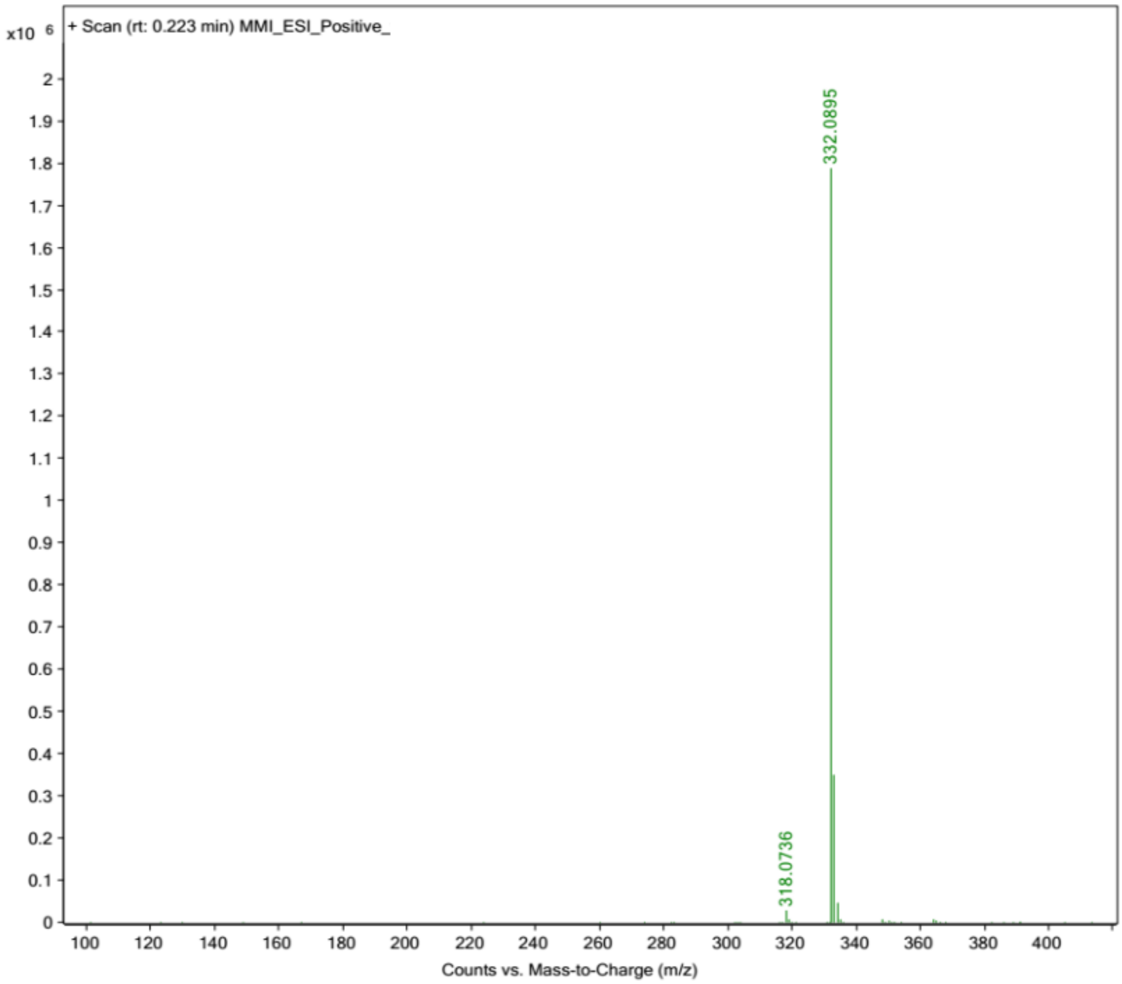

Figure S3 HRESIMS spectrum of compound **1**
